# Annotations

**Published:** 1895-08-31

**Authors:** 


### Annotations.

### The Death of Dr. J. S. Bristowe, F.R.S., LL.D.

By the death of Dr. Bristowe, English medicine loses one of its most cultured representatives, and English social life one of its most courteous and true-hearted gentlemen. As a man and as a physician Dr. Bristowe was of an ideal type. A physician must be gentle, and Dr. Bristowe was gentle; sincere, and he was sincere; of rare skill and experience, and his skill and experience were rare. Of no man can it be said with truth that he has no faults. Yet who ever heard a whisper of blame against Dr. Bristowe? Though so gentle and withal so courteous and kind to all, weakness was unknown to him His courage was as true as his gentleness. Those who have stood with him, as the present writer has, when powerful forces have been arrayed against him, know that when once he had taken up his position he could not be moved. As quietly as fighting might be done he would do it. But fight he would to the very last in what he deemed to be a righteous cause. A rare friend he was. Let him but once give his confidence, and it would not be withdrawn except on the most convincing proofs of unworthiness. It has been told elsewhere, and in many forms, how learned Dr. Bristowe was, how skilful as a physician and a man of science, how honoured by colleges and learned societies, how widely known by his work and writings in every civilised country. All this we need not repeat. To those who conduct this journal he was something more than all this. He was a friend who took his stand at the beginning of a long conflict; and although he could gain neither wealth nor fame by his loyalty, he never swerved to the last day of his life. By his death the Hospitals Association loses an honoured president, the Royal National Pension Fund its honorary medical adviser, the Royal College of Physicians its senior censor, and St. Thomas's Hospital its most distinguished consulting physician. Throughout the medical profession Dr. Bristowe will be mourned as a friend. Like so many other medical men of all ranks, he has died comparatively young. He had only reached the sixty-ninth year of his age.

### The Clothing of the Police.

Our sympathy goes out to the policemen who, during the recent hot weather, have had to do duty in their ordinary clothing. While the great peculiarity of our climate is its variability, that of the London policeman's clothing is, on the contrary, its unchanging sameness. Whether in blazing sun or in driving rain, in the heat of summer or the chills of winter, the policeman appears at the same hour, at the same point, at the same duty, and, except in regard to his top-coat, in the same clothes—and, of course, he suffers. The occasional bursts of tropical weather which sweep over London are, as is well known, productive of the extremest distress to such members of the police force as are on duty in places where shade is unattainable. Everyone else seeks comfort in loose clothing; but the policeman is allowed no such relief. However hot it may be, not only must he wear the same uniform, but it must be buttoned up to the neck in the same manner as in colder weather; and thus with his thick tunic, his tight belt, and his heavy helmet he literally stews—a victim to the idea of "smartness" in the semi-military sense which prevails at headquarters. There is no doubt that this is a matter in which reform is urgently required. At the same time, it must not be assumed, as it seems to be by some, that a separate summer uniform is what is wanted. The nights and mornings in summer are cool enough, and we know well that when the sun chooses to shine in March or April, the heat may be by no means inconsiderable. What is wanted is not a variety of uniforms so much as a power of varying the warmth of the uniform which is being worn. In civil life, when a man is cold, he buttons up his coat, and when he is too hot, he throws it open so that the air can freely permeate his clothing; and although it may not be possible to introduce such freedom into a policeman's uniform, this is the direction which reform should take. Adaptability of uniform to varying conditions would be far more valuable to the police than the provision of thinner clothing for fine days, for no one can tell what weather even the finest morning will bring forth.

### A Surgeon on Bicycles and Tricycles.

The number of middle-aged and elderly people who want to know all about cycling in these cycle-fever days is enormous. Nobody can tell them all they want to know better than a surgeon. Mr. Noble Smith, senior surgeon to the City Orthopædic Hospital, is himself a cyclist. He has been in the habit of recommending cycling for patients suffering from various weaknesses, whenever it has been desirable to take off the weight of the body from the legs; and he has been "well satisfied with the results." Concerning cycling generally, Mr. Noble Smith has the most decided opinions. For all whom it is suitable it is capital exercise. "Am I to ride a bicycle or a tricycle?" the middle-aged man wants to know. A bicycle, by all means, says Mr. Noble Smith, other things being equal. "A bicycle can be very quickly mastered without much risk of falls, even by a man past middle-age." Concerning the labour expended on the three-wheeled machine as compared with that demanded by the two-wheeler, there appears to be no room for differences of opinion. "The effort required to ride a tricycle is enormously greater than that needed for a bicycle; and the older and less agile the rider the more he will appreciate the difference." Even this does not exhaust the advantages claimed for the bicycle. For the two-wheeled machine a good track of a foot wide is sufficient, whilst the tricycle, as all tricyclists know, requires a three-foot track for comfortable action. In wet and windy weather, with dirty roads, tricycling is almost impossible. Not so with bicycling. This can be undertaken in almost any weather it is proper for men and women to be out in. The conclusion reached is, that cycling is good for almost all classes and ages; and that the bicycle is much to be preferred to the tricycle for the large majority of riders.

